# Supplementary material for: Non-Hermitian exceptional topology on a Klein bottle photonic circuit
Source: Light Sci Appl. 2026 Oct 21;15:390. doi: 10.1038/s41377-026-02516-x (PMC13687100; doi:10.1038/s41377-026-02516-x)
Supplement: Supplementary file 1 [file 41377_2026_2516_MOESM1_ESM.pdf]

Supplementary Information for Non-Hermitian Exceptional Topology on a Klein Bottle Photonic Circuit

Ze-Sheng Xu,[1,*] J. Lukas K. König,[2,*] Andrea Cataldo,[1] Rohan Yadgirkar,[1]
Govind Krishna,[1] Venkatesh Deenadayalan,[3] Val Zwiller,[1]
Stefan Preble,[3] Emil J. Bergholtz,[2] Jun Gao,[1,4,*]
Ali W. Elshaari[1,*]

[1]Department of Applied Physics, KTH Royal Institute of Technology, Albanova University Centre, Roslagstullsbacken 21, 106 91 Stockholm, Sweden
[2]Department of Physics, Stockholm University, AlbaNova University Center, 106 91 Stockholm, Sweden
[3]Microsystems Engineering, Rochester Institute of Technology, 1 Lomb Memorial Drive, Rochester, NY 14623
[4]School of Optical and Electronic Information, Huazhong University of Science and Technology, Luoyu Road 1037, Wuhan, Hubei, 430074, China
[*]E-mail: zesheng@kth.se, lukas.konig@fysik.su.se, jungao@hust.edu.cn, elshaari@kth.se

S1 Device Characterization and Calibration

The programmable photonic integrated circuit (PIC) under investigation is an 8×8 Mach-Zehnder interferometer (MZI) mesh fabricated on a silicon platform, designed for a central operating wavelength of 1550 nm. In this part we define the characterization process as determining the $I - V$ curve for every single heating resistance, and the calibration process as standardizing the working curve of each MZI unit.

S1.1 Characterization

In an ideal scenario, all thermo-resistors employed for on-chip thermal tuning should exhibit identical resistance values. In reality, however, their current-voltage characteristics inevitably differ due to fabrication imperfections and material inhomogeneities. To achieve accurate and independent control of each tuning element, it is essential to experimentally characterize their electrical properties prior to operation. The relationship between the applied voltage and the resulting current is not perfectly linear, a behavior that can be attributed to the change in resistance as the temperature of the heater increases. To accurately model this effect, a quadratic function fitting model was employed to describe the relationship between voltage and current:

$$U = IR_1 + I^2 R_2 \tag{S1}$$

In this model, R_1 represents the static resistance, while the term IR_2 accounts for the change in resistance caused by the self-heating effect, where R_2 is a positive constant ($R_2 > 0$). This nonlinear resistance

behavior is a crucial factor to consider for precise power and phase control. In a dense integrated mesh, thermal crosstalk between adjacent components is a significant concern. We use a special trench design on the silicon photonic chip to avoid the influence of thermal crosstalk as much as possible. After actual testing, the cross talk when applying current to different units is well within the acceptable error range.

S1.2 Calibration

The successful operation of the PIC as a fully programmable linear optical transformer capable of executing arbitrary unitary matrices hinges on an accurate and systematic calibration procedure. The central objective of the calibration is to construct explicit functional relations $\theta(I_\theta)$ and $\phi(I_\phi)$ for each node. This procedure guarantees that the experimentally realized MZI emulates the idealized MZI, with the calibrated phases accurately encoding both the internal and external phase-shifting elements of the mesh network. The process began with the internal phase shifters, which were calibrated using a Node Isolation Algorithm [1]. This algorithm is specifically designed to individually address each internal MZI node within the rectangular mesh structure, thereby eliminating the interference from other nodes during the calibration of a single one. The procedure starts by systematically isolating each node. This is achieved by launching light into a specific input port, and through careful control of the states of surrounding, already-calibrated nodes, the light is directed to pass exclusively through the MZI being calibrated before exiting at a single, designated output port. For example, by setting the adjacent MZI nodes to a "bar" state, the light path is constricted and directed straight to the MZI of interest. Once a node is isolated, we can then perform a voltage sweep on its internal phase shifter. By simultaneously monitoring the output optical power, we can accurately obtain the optical power-current curve for that specific component.

From this process, the precise function that maps current to phase shift, $\theta(I_\theta)$, is derived. Once these are calibrated, they are then set to a predefined state (e.g., either "bar" or "cross") to act as a routing element, which in turn isolates nearby uncalibrated nodes, allowing the calibration to move sequentially to the next diagonal, moving one diagonal at a time, from the top and bottom of the mesh towards the main diagonal, until every internal phase shifter has been individually and accurately calibrated. Following the completion of the internal phase shifter calibration, the external phase shifters can be calibrated. The calibration of the external phase shifter is performed using an interferometric splitting-recombination scheme [5]. The MZIs for splitting and recombination are both set to $\theta = \pi/2$, while the intermediate guiding MZIs are fixed at the bar state ($\theta = \pi$). By varying the current applied to the external phase shifter, a relative optical path difference between the upper and lower arms is introduced, resulting in constructive or destructive interference at the output. Recording the corresponding optical power as a function of the applied current yields the interference fringe, thereby completing the calibration of the external phase shifter. With the full set of calibration functions, $\theta(I_\theta)$ for internal shifters and $\phi(I_\phi)$ for external shifters, the entire mesh can be programmed with complex values.

Fig. S1 presents the fabricated device. Panel (a) shows a microscope image of the 8-mode device in the top panel, while the bottom panel illustrates the computational unit, implemented as a Mach–Zehnder interferometer (MZI). The MZI consists of two multimode interferometers (MMIs) connected by waveguides, with a total length of approximately 1120 μm. Panel (b) displays the optically and electrically packaged device, including fiber arrays for optical I/O and visible gold interconnects.

S1.3 Analysis of Loss Hierarchy and Its Impact on Hamiltonian Emulation

The integrity of emulating a non-Hermitian Hamiltonian on a PIC depends on the precise control of loss mechanisms. We categorize the optical losses in our platform into two hierarchies: mode-specific (path-dependent) losses and global losses.

1. **Global Insertion Loss:** The primary loss contribution from the fiber-to-chip interface (inverse tapers and fiber arrays) is around 4 dB. As these components are at the boundaries of the PIC, they

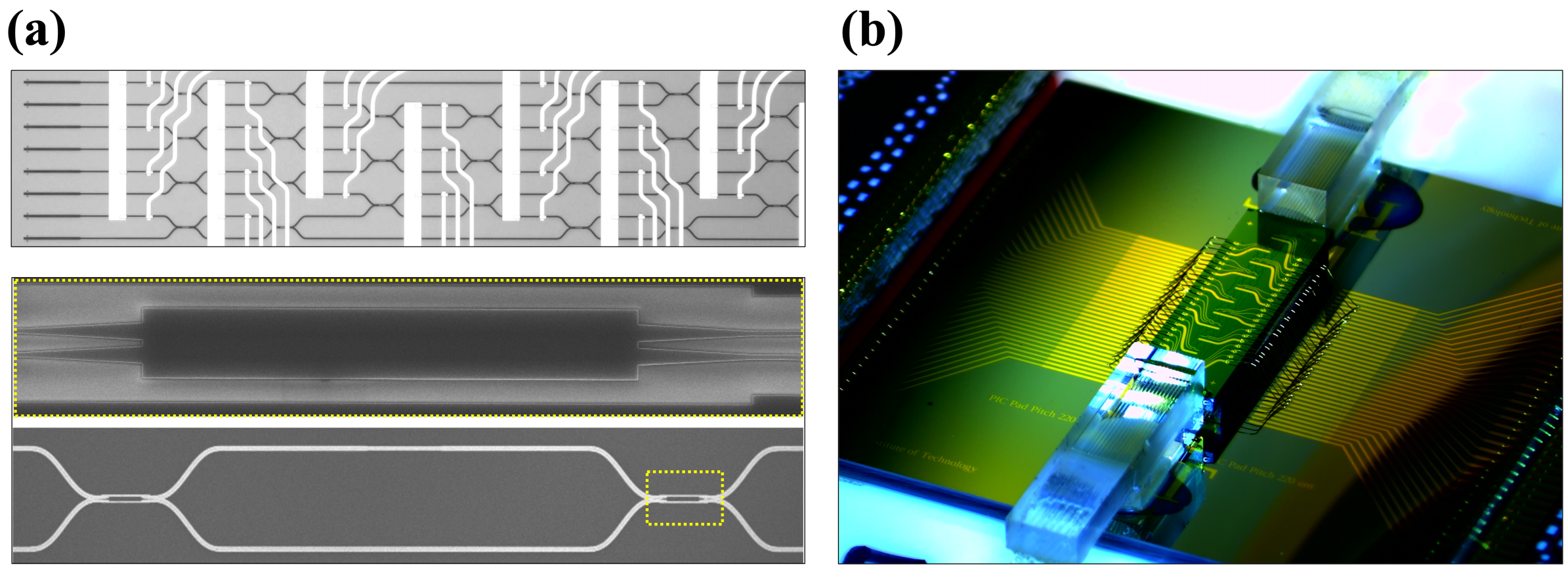

Figure S1: **Fabricated device.** (a) Microscope image of the 8-mode device and computational unit. (b) Packaged device with fiber arrays and gold interconnects. The arm length of the Mach–Zehnder interferometer (MZI) is 560 μm.

introduce a uniform attenuation that can be represented as a global identity term $-i\Gamma_{\text{global}}\mathbb{I}$, which does not alter the eigenstates or the exceptional topology of the system, as we show in Section S4.

2. **Intrinsic and Component Losses:** Internal losses consist of waveguide propagation loss ($<$ 2.5 dB cm^{-1} over about 1 cm device propagation length) and MMI insertion loss ($<$ 0.2 dB per unit). While non-uniformity in these localized losses could theoretically lead to experimental deviations, our systematic calibration of the 8×8 mesh confirms that path-dependent variations are negligible and remain well below the experimental error margin.

The maximum controlled attenuation achievable in our setup is designed to be significantly larger than any residual background loss variations across different spatial paths. Consequently, the effective Hamiltonian H' is dominated by the programmed parameters, effectively suppressing the impact of random chip imperfections on the observed non-Hermitian phenomena.

S2 Fidelity Estimation

This section outlines the procedure used to quantify the agreement between experimental measurements and numerical simulations. The goal is to obtain a robust fidelity metric for time-series data from the two output modes of the photonic circuit, accounting for both temporal waveform matching and relative mode intensities.

Data preprocessing

Simulation data consist of numerically generated intensity traces for modes 1 and 2, while the experimental data are acquired from the PIC using the rapid-switching detection scheme described in the main text. To enable direct comparison, both traces undergo normalization to remove scale differences while preserving temporal structure:

$$y_1 \mapsto \frac{y_1}{M}, \qquad y_2 \mapsto \frac{y_2}{M}, \qquad M = \max\{\max(y_1), \max(y_2)\}. \tag{S2}$$

For mode 2, this normalization also compensates for the calibrated insertion loss of the optical switch, ensuring that relative mode intensities reflect the physical PIC output rather than instrumental effects.

Correlation-based fidelity

Agreement between simulation and experiment for each mode is quantified using the Pearson correlation coefficient:

$$\rho = \frac{\sum_{i=1}^{n}(a_i - \bar{a})(b_i - \bar{b})}{\sqrt{\sum_{i=1}^{n}(a_i - \bar{a})^2}\sqrt{\sum_{i=1}^{n}(b_i - \bar{b})^2}}, \tag{S3}$$

where $\{a_i\}$ and $\{b_i\}$ denote simulated and measured samples, respectively. This yields two correlation values, one per mode, describing the temporal match between theory and experiment.

Intensity-based weighting

Because the two output modes generally carry different total intensities, we assign weights proportional to their relative contributions:

$$\eta_i = \frac{\bar{b}_i}{\bar{b}_1 + \bar{b}_2}, \tag{S4}$$

where $\bar{b}_i$ is the mean experimental intensity of mode i. The weights η_1 and η_2 sum to unity, ensuring that the final fidelity metric reflects the physical significance of each mode.

Final fidelity metric

The full fidelity is computed as a weighted sum of the two correlation coefficients and constrained to the range $[0, 1]$:

$$F = \eta_1\rho_1 + \eta_2\rho_2.$$

This metric jointly captures (i) temporal alignment of oscillations and decay features, and (ii) correct relative scaling between the two modes.

Validation

The methodology was validated using synthetic datasets with controlled noise and known correlation structure. The weighted approach proved more robust to uneven noise levels and imbalanced mode amplitudes than unweighted correlation metrics. When applied to the experimental data of the main text, all tested parameter points exhibited fidelities exceeding 95%, confirming the excellent agreement between the implemented non-Hermitian dynamics and theoretical predictions.

S3 Intensity Measurements and Decay-Rate Analysis

To characterize the system dynamics, we measure the time evolution of several initial states injected into the two-mode subsystem:

$$A_1(0) = \frac{[1, i]^{\mathrm{T}}}{\sqrt{2}}, \qquad A_2(0) = \frac{[1, -i]^{\mathrm{T}}}{\sqrt{2}}, \qquad A_3(0) = [1, 0]^{\mathrm{T}}, \tag{S5}$$

which correspond to distinct points on the Bloch sphere, as illustrated in Fig. S2a.

Our primary interest lies in detecting the bulk Fermi arc of the Hamiltonian introduced in the main text, since the winding numbers and exceptional topology are entirely encoded in the locations where the

imaginary parts of the eigenvalues become degenerate. Importantly, the goal here is not to reconstruct the full eigenstructure but to provide a qualitative method for identifying whether a parameter point lies on the imaginary Fermi arc. This can be achieved using intensity-only measurements, without requiring phase estimation.

A generic initial state $|\psi_0\rangle$ evolves under a (non-Hermitian) Hamiltonian H as:

$$e^{-iHt}|\psi_0\rangle = c_1 e^{-i\lambda_1 t}|v_1\rangle + c_2 e^{-i\lambda_2 t}|v_2\rangle, \tag{S6}$$

where the c_i are overlaps with eigenvectors $|v_i\rangle$ and λ_i are the corresponding complex eigenvalues. If $\mathrm{Im}(\lambda_1) = \mathrm{Im}(\lambda_2)$, the total intensity decays at the same rate for all initial states. Conversely, a difference in imaginary parts produces distinct decay behaviors depending on the relative magnitudes of c_1 and c_2.

Ideally, one could excite the eigenvectors directly, but this is impossible without already knowing the eigenstructure. Instead, we compare intensity evolution for several generic initial states. Using only a single input state can lead to false positives (e.g. when $|c_1| \approx |c_2|$), so we employ three non-parallel states to avoid this degeneracy.

Fig. S2b–c illustrates this approach. In panel (b), the chosen parameter point lies on the Fermi arc: all three input states decay at nearly identical rates, indicating $\mathrm{Im}(\lambda_1) = \mathrm{Im}(\lambda_2)$. In panel (c), corresponding to an off-arc point, the decay rates differ appreciably among the three initial states, signaling a finite imaginary-eigenvalue splitting.

To quantify such differences, we define the bifurcation parameter

$$\delta = \max_{i,j} \mathrm{Var}\big(I_i - I_j\big), \tag{S7}$$

where I_i and I_j denote the total intensities obtained from different input states. A small value of δ indicates nearly identical decay traces (on the Fermi arc), while a large value reflects divergence (off the arc).

Fig. S3 shows the phase diagram of δ across the (p, q) plane for different global rotation factors applied to the Hamiltonian,

$$H' = e^{-i\theta}H, \tag{S8}$$

demonstrating good agreement between theoretical predictions and experimental measurements. The left column shows the imaginary components of δ for rotation angles between 0 and 0.8π, while the right column displays the corresponding real parts.

Although intensity-only measurements can reliably indicate whether a point is on or off the Fermi arc, their resolution is ultimately limited by noise, switching drift, and other experimental uncertainties. In the next section, we show that incorporating phase-sensitive measurements enables full reconstruction of the complex evolution operator and thereby provides a far more accurate and noise-resistant map of the Fermi arc, at the cost of significantly longer measurement and data-processing time.

S4 Invariance of Eigenstates under Global Loss Transformations

To provide a rigorous demonstration for a broader audience, we detail the mathematical equivalence between the target non-Hermitian Hamiltonian H and the experimentally implemented pure-loss Hamiltonian H'. In our work, we employ a differential-loss scheme where the evolution is governed by $H' = H - i\Lambda\mathbb{I}$. Here, $\Lambda > 0$ is a global decay constant chosen to ensure that all eigenvalues lie in the lower-half complex plane, making the system physically realizable using only loss mechanisms.

Consider the eigenvalue problem for the original non-Hermitian Hamiltonian H:

$$H|\psi_n\rangle = E_n|\psi_n\rangle \tag{S9}$$

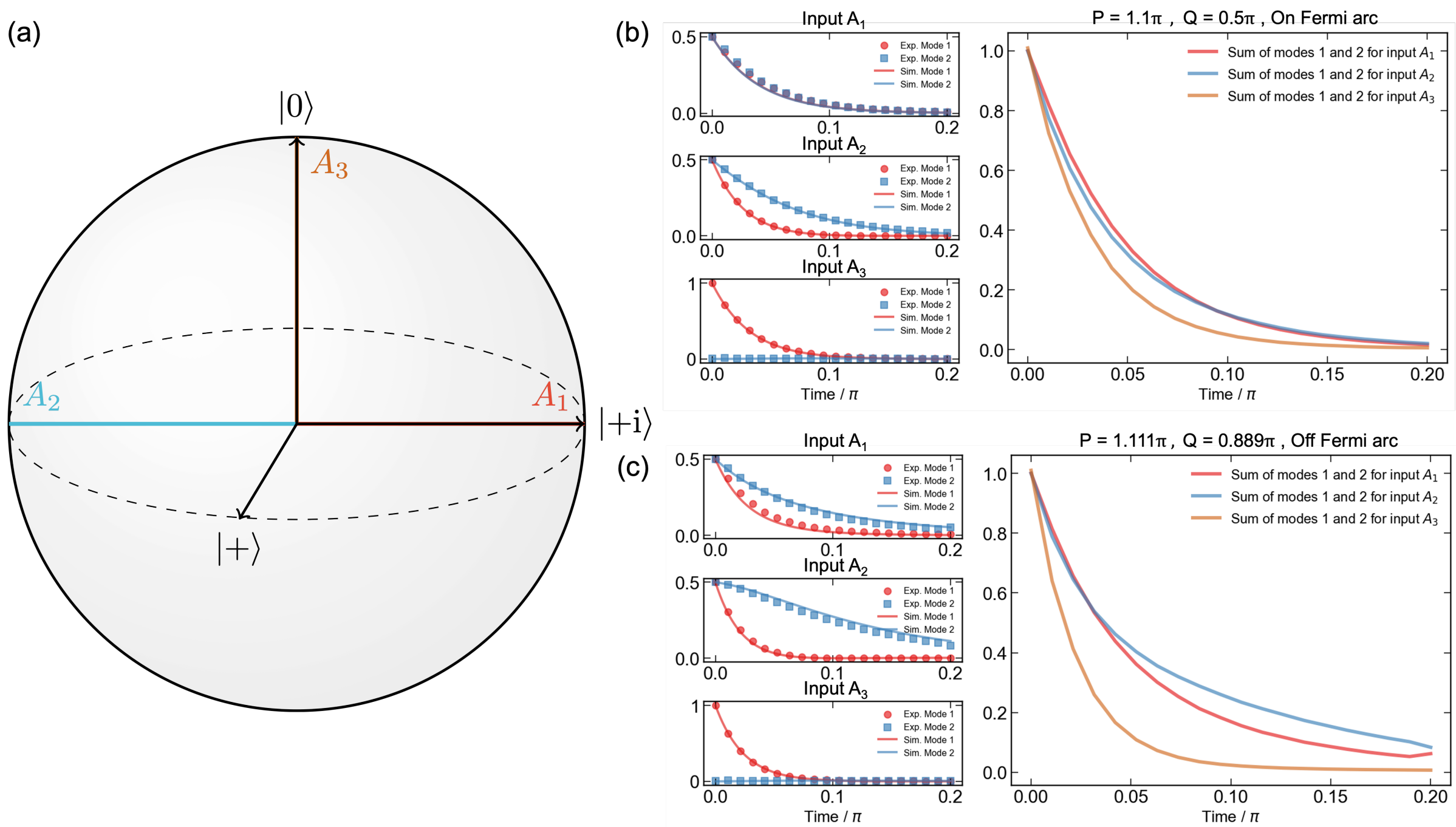

Figure S2: **Experimental and simulated dynamics on and off the imaginary Fermi arc.** **(a)** Bloch sphere representation of the three selected input states, A_1, A_2, and A_3, which are injected into the non-Hermitian Klein-bottle Hamiltonian described in the main text. **(b)** *On the Fermi arc:* results for the parameter point $(p, q) = (1.1\pi,\ 0.5\pi)$. The three panels on the left show the experimentally measured intensity evolution in modes 1 and 2 (discrete data points) together with the corresponding theoretical simulations (solid lines), demonstrating excellent agreement. The right panel displays the total intensity, obtained by summing modes 1 and 2, for the three input vectors indicated in (a). **(c)** *Off the Fermi arc:* results for the parameter point $(p, q) = (1.111\pi,\ 0.889\pi)$. The same comparison structure is used as in (b). In contrast to the on-arc case, the total intensity exhibits a clear bifurcation, confirming the transition from degenerate to non-degenerate imaginary eigenvalues.

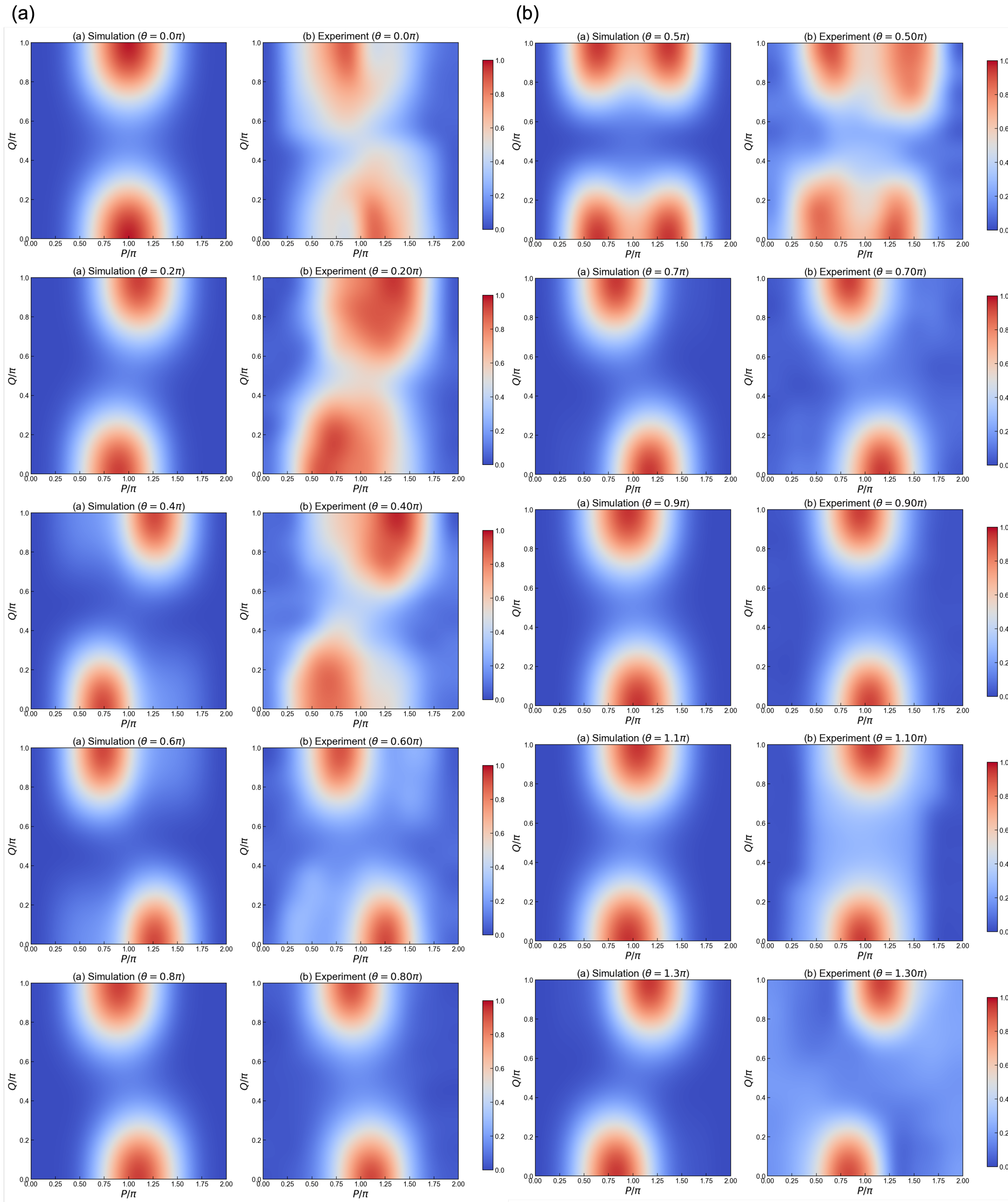

Figure S3: **Comparison between simulation and experimental maps probing the phase diagram. (a)** Pairwise variance maps to quantify the bifurcation for different input states corresponding to those shown in Fig. S2. The left column shows the simulated results and the right column the experimental measurements, for angles in the Hamiltonian $\theta = 0\pi$, 0.2π, 0.4π, 0.6π, and 0.8π, which probe the *imaginary phase diagram*. In total, 100 experiments (a 10×10 grid) were performed to map the (p, q) parameter space of the Hamiltonian. **(b)** Corresponding pairwise variance maps obtained when multiplying the Hamiltonian by $1j$, allowing access to the *real phase diagram*. The measurements and simulations are shown for $\theta = 0.5\pi$, 0.7π, 0.9π, 1.1π, and 1.3π, showing good agreement between experiment and theory.

where E_n and $|\psi_n\rangle$ represent the n-th eigenvalue and the corresponding eigenstate (right eigenvector), respectively. When we construct the pure-loss Hamiltonian H' by introducing the global decay factor $-i\Lambda\mathbb{I}$, the action of H' on the original eigenstate $|\psi_n\rangle$ is given by:

$$H'|\psi_n\rangle = (H - i\Lambda\mathbb{I})|\psi_n\rangle = H|\psi_n\rangle - i\Lambda|\psi_n\rangle \tag{S10}$$

By substituting the original eigenvalue equation into the above expression, we obtain:

$$H'|\psi_n\rangle = (E_n - i\Lambda)|\psi_n\rangle \tag{S11}$$

This result explicitly demonstrates that $|\psi_n\rangle$ remains an eigenstate of the modified Hamiltonian H'. The transformation only results in a uniform shift of the complex eigenvalues: $E_n \to E_n - i\Lambda$.

The time evolution operator for the system is $T(t) = e^{-iH't}$. Consequently, the state at time t evolves as:

$$|\Psi(t)\rangle = e^{-i(H-i\Lambda\mathbb{I})t}|\Psi(0)\rangle = e^{-\Lambda t}e^{-iHt}|\Psi(0)\rangle \tag{S12}$$

This indicates that the non-unitary dynamics governed by H' preserve the identical modal structures and topological features—such as Fermi arcs and exceptional points—as the original system H. The experimental intensities measured at time t differ from the theoretical model only by a global exponential decay factor $e^{-2\Lambda t}$ (or $e^{-\Lambda t}$ for field amplitudes), which is numerically compensated during data post-processing to retrieve the intrinsic non-Hermitian dynamics.

S5 4×4 Unitary Dilation Using Ancillary Modes

S5.1 Overview and Motivation

A central challenge in emulating non-Hermitian dynamics on a photonic integrated circuit is that the time-evolution operator $T(t) = e^{-iHt}$ is **non-unitary** whenever H is non-Hermitian. Physical optical systems governed by passive linear optics, however, implement **unitary** transformations that conserve total photon number. To bridge this gap, we employ a **unitary dilation** technique [8]: the 2×2 non-unitary operator T is embedded as a subblock of a larger 4×4 unitary matrix, with the additional degrees of freedom provided by **ancillary modes** that couple to the environment.

The target non-unitary operator has the general form:

$$T = \begin{pmatrix} T_{00} & T_{01} \\ T_{10} & T_{11} \end{pmatrix}. \tag{S13}$$

Our goal is to construct a 4×4 unitary $U^{(4)}$ such that when light is injected into modes 2 and 3 (the "system" modes), and we discard the output from modes 1 and 4 (the "ancilla" modes), the effective transformation on the system subspace is exactly T.

S5.2 Takagi Factorization for Complex-Symmetric Matrices

The first step is to decompose T into a form suitable for physical implementation. For a general complex matrix, the standard tool is the **singular value decomposition (SVD)**:

$$T = V\Sigma W^\dagger, \tag{S14}$$

where V and W are unitary and Σ is diagonal with non-negative real entries (the singular values).

However, the Hamiltonian in our experiment has a special structure. It is **complex symmetric**:

$$H = \begin{pmatrix} ia & c \\ c & ib \end{pmatrix}, \qquad a, b, c \in \mathbb{R}, \tag{S15}$$

satisfying $H = H^{\mathrm{T}}$ (but $H \neq H^{\dagger}$, so it is non-Hermitian). The time-evolution operator $T = e^{-iHt}$ inherits this symmetry: $T = T^{\mathrm{T}}$.

For complex-symmetric matrices, a more efficient decomposition exists—the **Autonne–Takagi factorization** [2, 10]:

$$T = VDV^{\mathrm{T}}, \tag{S16}$$

where:

- V is a unitary matrix,
- $D = \mathrm{diag}(D_1, D_2)$ is a diagonal matrix with **real, non-negative** entries.

This factorization is advantageous because it requires only **one** unitary V (rather than two distinct unitaries as in SVD), simplifying the optical implementation.

S5.3 Physical Interpretation of the Dilation

The diagonal matrix D contains the **singular values** of T. Since T is non-unitary (representing a system with gain or loss), we have $D_1, D_2 \leq 1$ for a purely lossy system. The "missing" amplitude, $\sqrt{1 - D_i^2}$, represents the fraction of light that must be **diverted to ancilla modes** to account for the non-unitary evolution.

We construct normalized amplitude vectors that distribute light between system and ancilla modes:

$$D_{\mathrm{m1}} = \begin{pmatrix} \sqrt{1 - D_1^2} \\ D_1 \end{pmatrix}, \qquad D_{\mathrm{m2}} = \begin{pmatrix} D_2 \\ \sqrt{1 - D_2^2} \end{pmatrix}. \tag{S17}$$

Here:

- D_i is the amplitude remaining in the system mode,
- $\sqrt{1 - D_i^2}$ is the amplitude redirected to the ancilla.

These vectors are unit-normalized by construction, ensuring energy conservation in the dilated unitary.

S5.4 MZI-Based Implementation

Each Mach–Zehnder interferometer (MZI) in our mesh implements a 2×2 unitary transformation:

$$U_{\mathrm{MZI}}(\theta, \phi) = e^{i(\theta+\pi)/2} \begin{pmatrix} e^{i\phi} \sin(\theta/2) & \cos(\theta/2) \\ e^{i\phi} \cos(\theta/2) & -\sin(\theta/2) \end{pmatrix}. \tag{S18}$$

The parameters θ and ϕ are controlled by **internal** and **external** thermo-optic phase shifters, respectively:

- θ controls the **splitting ratio** (how much light goes to each output),
- ϕ controls the **relative phase** between the two arms.

Generating the amplitude vectors:
To produce D_{m1} from the input state $(1, 0)^{\mathrm{T}}$, we configure an MZI followed by a phase shifter:

$$\begin{pmatrix} e^{i\phi_2} & 0 \\ 0 & 1 \end{pmatrix} U_{\mathrm{MZI}}(\theta, \phi_1) \begin{pmatrix} 1 \\ 0 \end{pmatrix} = D_{\mathrm{m1}}. \tag{S19}$$

Setting $\phi_1 = 0$ for simplicity, we solve for the required angles:

$$\theta = 2\arcsin(D_1), \qquad \phi_2 = -\frac{\theta + \pi}{2}. \tag{S20}$$

An analogous procedure yields the parameters (θ', ϕ_2') for generating D_{m2} from the input $(0,1)^{\mathrm{T}}$. We define the resulting 2×2 blocks:

$$D_{\mathrm{up}} = \begin{pmatrix} e^{i\phi_2} & 0 \\ 0 & 1 \end{pmatrix} U_{\mathrm{MZI}}(\theta, 0), \qquad D_{\mathrm{low}} = \begin{pmatrix} e^{i\phi_2'} & 0 \\ 0 & 1 \end{pmatrix} U_{\mathrm{MZI}}(\theta', 0). \tag{S21}$$

S5.5 Assembling the 4×4 Unitary

The full 4×4 dilation is constructed from three components acting on the four-mode space (ancilla 1, system mode 1, system mode 2, ancilla 2):

1. Input unitary $U_2^{(4)}$: Applies V to the system modes while leaving ancillas unchanged:

$$U_2^{(4)} = I_1 \oplus V \oplus I_1 \tag{S22}$$

2. Diagonal attenuation block $D^{(4)}$: Couples each system mode to its corresponding ancilla with the appropriate amplitude ratio:

$$D^{(4)} = D_{\mathrm{up}} \oplus D_{\mathrm{low}} \tag{S23}$$

3. Output unitary $U_1^{(4)}$: Applies V^{T} to the system modes:

$$U_1^{(4)} = I_1 \oplus V^{\mathrm{T}} \oplus I_1 \tag{S24}$$

The complete 4×4 unitary is:

$$U^{(4)} = U_1^{(4)} \cdot D^{(4)} \cdot U_2^{(4)}. \tag{S25}$$

Verification: When we inject light into the system modes (modes 2 and 3) and trace out the ancilla modes (modes 1 and 4), the effective transformation on the system subspace is:

$$T_{\mathrm{eff}} = V D V^{\mathrm{T}} = T, \tag{S26}$$

where the last equality uses $D = D^{\mathrm{T}}$ (diagonal matrices are symmetric).

S5.6 Embedding in the Full Mesh

The 4×4 dilation block is embedded within the 8×8 programmable mesh by setting all other modes to pass through unchanged:

$$U^{(8)} = U^{(4)} \oplus I_4. \tag{S27}$$

In practice, this means configuring the MZIs outside the active 4×4 region to the **bar state** ($\theta = \pi$), so light in those modes propagates straight through without mixing.

S5.7 From Parameter Space to Hardware: Algorithmic Synthesis

To experimentally realize the non-Hermitian Hamiltonian at a specific point (p, q) in the Klein-bottle parameter space, we follow a deterministic multi-layer mapping procedure:

Layer 1: Parameter to Hamiltonian. Given coordinates (p, q), we construct the theoretical Hamiltonian according to Eq. (4) of the main text:

$$H(p,q) = \begin{pmatrix} -a_{p,q} & c \\ c & a_{p,q} \end{pmatrix}, \tag{S28}$$

with $a_{p,q} = \sin p \cos q + i(1 - \cos p)(1 + \cos(2q)/2)$.

Layer 2: Hamiltonian to Evolution Operator. For a chosen evolution time t_n, we compute the non-unitary time-evolution operator:

$$T(t_n) = e^{-iH(p,q)t_n}. \tag{S29}$$

Layer 3: Evolution Operator to Unitary Dilation. We perform the Takagi decomposition $T = VDV^{\mathrm{T}}$ and construct the 4×4 (or 6×6 for phase measurements) unitary dilation as described above.

Layer 4: Unitary to MZI Parameters. The target unitary $U^{(4)}$ is decomposed into a product of MZI operations. Each MZI requires two phase settings (θ, ϕ), which are computed analytically from the matrix decomposition.

Layer 5: MZI Parameters to Electrical Currents. Finally, the target phases are converted to electrical currents using the calibration curves established in Section S1:

$$\Delta\phi = \alpha I^2 + \beta, \tag{S30}$$

where α and β are heater-specific calibration constants.

This hierarchical workflow—from abstract topological coordinates to physical electrical signals—enables precise, reproducible navigation of the Klein-bottle parameter space without requiring any iterative optimization or feedback loops. It is also noteworthy that while other pioneering platforms realize non-Hermitian landscapes by precisely tuning physical degrees of freedom, such as the observation of polarizations based on fibre-loop structure [6] or structural 3D chirality in a metasurface [3], our approach utilizes a programmable digital-analog hybrid mapping that provides unique flexibility for exploring arbitrary parameter spaces.

S6 6×6 Unitary Dilation for Phase Measurement and Hamiltonian Retrieval

S6.1 Overview and Motivation

The 4×4 dilation described in the previous section allows us to measure the **amplitudes** $|T_{ij}|$ of the non-unitary evolution operator. However, to fully reconstruct the Hamiltonian and map the Fermi arc, we also need the **complex phases** $\arg(T_{ij})$ of each matrix element.

Measuring phases in photonics is fundamentally more challenging than measuring intensities. Standard approaches require **heterodyne detection** with external local oscillators and balanced photodiodes. Here, we circumvent this complexity by exploiting the programmability of the MZI mesh itself: we construct a 6×6 unitary dilation that enables **on-chip interferometric phase extraction** using only intensity measurements.

The key idea is to interfere each unknown matrix element T_{ij} with a known reference amplitude. By scanning the reference phase and fitting the resulting intensity fringes, we extract the phase of T_{ij} without any external coherent detection hardware.

S6.2 Review: Measuring Field Amplitudes

At a discrete evolution time t, the two-mode system evolves under the non-unitary operator:

$$T(t) = e^{-iHt} = \begin{pmatrix} T_{00} & T_{01} \\ T_{10} & T_{11} \end{pmatrix}. \tag{S31}$$

To measure the amplitudes $|T_{00}|$, $|T_{01}|$, $|T_{10}|$, $|T_{11}|$, we inject the canonical basis states:

$$e_0 = \begin{pmatrix} 1 \\ 0 \end{pmatrix}, \qquad e_1 = \begin{pmatrix} 0 \\ 1 \end{pmatrix}, \tag{S32}$$

and measure the output intensities at both ports. For example:

- Injecting e_0 and measuring port 1 gives $|T_{00}|^2$,
- Injecting e_0 and measuring port 2 gives $|T_{10}|^2$,
- Injecting e_1 and measuring port 1 gives $|T_{01}|^2$,
- Injecting e_1 and measuring port 2 gives $|T_{11}|^2$.

Since $T(t)$ is non-unitary, we access it experimentally through a unitary dilation. The standard dilation embeds T into a 4×4 unitary:

$$W = \begin{pmatrix} T & D_{T^\dagger} \\ D_T & -T^\dagger \end{pmatrix}, \qquad D_T = \sqrt{\mathbb{I} - T^\dagger T}. \tag{S33}$$

Here, D_T is the **defect matrix** that quantifies how much T deviates from unitarity. By injecting light into the first two modes (system modes) and measuring outputs at those same modes, we obtain the amplitudes of T.

S6.3 The Phase Extraction Problem

Amplitudes alone do not fully characterize $T(t)$. Each matrix element is a complex number:

$$T_{ij} = |T_{ij}| e^{i\theta_{ij}}, \tag{S34}$$

where $\theta_{ij} = \arg(T_{ij})$ is the phase we need to extract.

The challenge is that photodetectors measure only intensity $I = |E|^2$, which is insensitive to absolute phase. To recover phase information, we must convert phase differences into intensity variations through **interference**.

The principle is straightforward: if we interfere an unknown field $E_{\text{unknown}} = |E|e^{i\theta}$ with a known reference field $E_{\text{ref}} = Re^{i\phi}$, the output intensity is:

$$I(\phi) = |E_{\text{unknown}} + E_{\text{ref}}|^2 = |E|^2 + R^2 + 2|E|R\cos(\theta - \phi). \tag{S35}$$

By scanning the reference phase ϕ and fitting the resulting intensity fringe, we extract the unknown phase θ.

S6.4 Constructing the 6×6 Interference Matrix

To implement on-chip interference, we extend the 4×4 dilation to a 6×6 structure by adding an auxiliary **interference block**. This block mixes the unknown matrix element with a programmable reference.

For a matrix element $E_i \equiv T_{ij}$ whose phase we wish to measure, we construct an auxiliary 2×2 unitary:

$$\mathcal{M}_{\text{aux}} = \begin{pmatrix} -E_i & R_i \\ R_i & E_i^* \end{pmatrix}, \qquad R_i = \sqrt{1 - |E_i|^2}. \tag{S36}$$

This matrix has a specific structure:

- The diagonal elements involve the unknown E_i and its conjugate E_i^*,

- The off-diagonal elements are real and equal to R_i, which serves as the reference amplitude,
- The matrix is unitary by construction: $\mathcal{M}_{\text{aux}}^{\dagger}\mathcal{M}_{\text{aux}} = \mathbb{I}$.

The full 6×6 interference matrix combines this auxiliary block with the original 4×4 dilation:

$$\mathcal{U}^{(6)} = \begin{pmatrix} \mathcal{M}_{\text{aux}} & 0 \\ 0 & W \end{pmatrix}. \tag{S37}$$

This block-diagonal structure ensures that the interference block operates independently of the main dilation, while both are implemented within the same 8×8 mesh.

S6.5 Generating Orthogonal Probe States

To extract phases reliably, we need to probe the system along multiple linearly independent directions. Although light enters through a single physical input port, we use the programmable MZI mesh to transform the input into different **probe states**.

Starting from the canonical input vector:

$$E_0 = (1, 0, 0, 0, 0, 0)^{\text{T}}, \tag{S38}$$

we apply transformation operators Π_i to generate orthogonal probe states:

$$v_i = \Pi_i E_0. \tag{S39}$$

A convenient choice of probe vectors that provides sensitivity to all matrix element phases is:

$$v_1 = \begin{pmatrix} 0 \\ \frac{1}{\sqrt{2}}e^{i\phi} \\ \frac{1}{\sqrt{2}} \\ 0 \\ 0 \\ 0 \end{pmatrix}, \qquad v_2 = \begin{pmatrix} 0 \\ \frac{1}{\sqrt{2}}e^{i\phi} \\ 0 \\ \frac{1}{\sqrt{2}} \\ 0 \\ 0 \end{pmatrix}. \tag{S40}$$

The tunable phase ϕ in these probe states is the key to phase extraction. When v_1 propagates through the 6×6 matrix $\mathcal{U}^{(6)}$, the output intensity depends on the interference between:

- The unknown matrix element $E_i e^{i\theta}$ (carrying the phase we want to measure),
- The reference component $R_i e^{i\phi}$ (with the known, scannable phase).

The resulting intensity takes the form:

$$I(\phi) = |E_i e^{i\theta} + R_i e^{i\phi}|^2 = |E_i|^2 + R_i^2 + 2|E_i|R_i \cos(\theta - \phi). \tag{S41}$$

By scanning ϕ from 0 to 2π and recording the intensity fringe, we fit to this functional form and extract the unknown phase θ.

S6.6 Mode Routing with Switching Matrices

Different matrix elements T_{ij} correspond to different spatial paths through the MZI mesh. To route light appropriately for measuring each element, we introduce **switching matrices** that reconfigure the internal mode connectivity.

We define two switching matrices:

$$s_1 = \frac{1}{\sqrt{2}} \begin{pmatrix} 1 & 1 \\ 1 & -1 \end{pmatrix}, \qquad s_2 = \frac{1}{\sqrt{2}} \begin{pmatrix} 1 & 0 & 1 \\ 0 & \sqrt{2} & 0 \\ 1 & 0 & -1 \end{pmatrix}. \tag{S42}$$

These are embedded into the 6×6 space as:

$$S_j = I_1 \oplus s_j \oplus I_k, \tag{S43}$$

where the identity matrices I_1 and I_k act on the modes not involved in the switching.

The switching matrices serve the following purposes:

- S_1 is applied when extracting phases of T_{00} and T_{01} (first row of T),
- S_2 is applied when extracting phases of T_{10} and T_{11} (second row of T).

S6.7 Complete Phase Extraction Protocol

Combining all components, the complete output field for a given probe state and switching configuration is:

$$\mathcal{O}(t) = S_j \cdot \mathcal{U}^{(6)} \cdot \Pi_i \cdot E_0, \tag{S44}$$

and the measured intensity is $I = |\mathcal{O}(t)|^2$.

The phase extraction protocol proceeds as follows:

Step 1: Amplitude measurement. Using the 4×4 dilation, measure $|T_{ij}|$ for all four matrix elements as described in Sections S6.2 and S6.3.

Step 2: Configure interference block. For each matrix element T_{ij}, program the auxiliary block $\mathcal{M}_{\text{aux}}$ using the measured amplitude $|T_{ij}|$.

Step 3: Phase scanning. For each probe state v_i:

- Scan the reference phase ϕ over the range $[0, 2\pi]$,
- Record the output intensity $I(\phi)$ at each step,
- Fit the intensity fringe to extract the phase θ_{ij}.

Step 4: Consistency check. Use multiple probe states to overdetermine the system. The unique set of phases consistent with all measurements is selected as the final result.

Because the PIC provides only intensity measurements, and because fabrication tolerances introduce small deviations in realized amplitudes, the fitting procedure is essential for robust phase recovery.

S6.8 Hamiltonian Reconstruction from Time-Evolution Data

To reconstruct the full Hamiltonian $H(p, q)$ at each point in parameter space, we combine amplitude and phase measurements across multiple evolution times.

Stage 1: Multi-time sampling. For each parameter point (p, q), we measure the complex evolution operator $T(t_n)$ at five discrete evolution times:

$$t_n \in \{0.05\pi,\ 0.10\pi,\ 0.15\pi,\ 0.20\pi,\ 0.25\pi\}. \tag{S45}$$

Multiple time points provide redundancy and improve fitting accuracy, especially in the presence of experimental noise.

Stage 2: Global fitting. The measured complex matrix elements $T_{ij}(t_n)$ across all time points are jointly fitted to the theoretical model:

$$T(t) = e^{-iHt}, \tag{S46}$$

using a nonlinear least-squares procedure. The fitting parameters are the real and imaginary parts of the Hamiltonian matrix elements.

Stage 3: Eigenvalue computation. Once $H(p, q)$ is reconstructed, we compute its complex eigenvalues:

$$H(p, q) |\psi_\pm\rangle = \lambda_\pm(p, q) |\psi_\pm\rangle . \tag{S47}$$

Stage 4: Fermi arc identification. The imaginary Fermi arc is identified as the locus of points where:

$$\mathrm{Im}(\lambda_+) = \mathrm{Im}(\lambda_-). \tag{S48}$$

This condition corresponds to equal decay rates for both eigenmodes, which produces the characteristic bifurcation signatures observed in the intensity measurements of Section S3.

S6.9 Summary of the Two-Stage Reconstruction Protocol

The complete Hamiltonian reconstruction protocol can be summarized as:

1. 4×4 **dilation:** Measure amplitudes $|T_{ij}(t_n)|$ at five evolution times.
2. 6×6 **dilation:** Measure phases $\arg(T_{ij}(t_n))$ via interferometric scanning.
3. **Matrix assembly:** Combine amplitudes and phases to form the complex matrix $T(t_n)$ at each time point.
4. **Hamiltonian fitting:** Fit $T(t_n) = e^{-iHt_n}$ across all time points to extract $H(p, q)$.
5. **Eigenvalue analysis:** Diagonalize $H(p, q)$ and compute $\mathrm{Im}(\lambda_\pm)$ to map the Fermi arc.

This two-stage approach—separating amplitude and phase measurements—provides a systematic and robust method for full Hamiltonian tomography using only on-chip intensity detection.

S6.10 Definition and Calculation of Hamiltonian Reconstruction Fidelity

To quantitatively evaluate the accuracy of our experimental Hamiltonian reconstruction protocol, we introduce a fidelity metric, $\mathcal{F}$. This metric measures the degree of agreement between the experimentally reconstructed effective Hamiltonian, H_{exp}, and the ideal theoretical Hamiltonian, H_{th}, at each targeted coordinate in the (p, q) parameter space.

We quantify the distance between the experimental and theoretical Hamiltonians using the Frobenius norm. For a general 2×2 complex matrix A, the Frobenius norm is defined as:

$$||A||_{\mathrm{F}} = \sqrt{\sum_{i=1}^{2}\sum_{j=1}^{2} |A_{ij}|^2}$$

The reconstruction fidelity $\mathcal{F}$ is then calculated by normalizing the Frobenius norm of the difference matrix, $\Delta H = H_{\mathrm{exp}} - H_{\mathrm{th}}$, against the sum of the norms of the individual matrices:

$$\mathcal{F} = 1 - \frac{||H_{\mathrm{exp}} - H_{\mathrm{th}}||_{\mathrm{F}}}{||H_{\mathrm{th}}||_{\mathrm{F}} + ||H_{\mathrm{exp}}||_{\mathrm{F}}}$$

This normalization ensures that $\mathcal{F}$ provides a bounded, dimensionless measure of similarity. A fidelity of $\mathcal{F} = 1$ indicates perfect agreement between theory and experiment, while deviations from 1 quantify the accumulated effects of experimental noise and fitting errors.

As referenced in the main text (Fig. 4b), the reconstructed diagonal terms for the topological point ($p = 0.5\pi, q = 0.5\pi$) exhibit minor non-zero real parts ($H^{\text{Re}}_{\text{exp},00} \approx -0.061$, $H^{\text{Re}}_{\text{exp},11} \approx 0.033$). These small deviations stem from inevitable residual experimental noise, such as slight thermal crosstalk and minor calibration imperfections during the time-evolution operator measurements. However, utilizing the rigorous metric defined above, the overall reconstruction fidelity for this specific point is calculated to be 98.83%. This high fidelity confirms that the structural integrity and the essential non-Hermitian properties of the Hamiltonian are highly preserved despite the presence of physical noise.

Furthermore, consistently high fidelities are observed across all sampled parameter points, validating the robustness of our optical mesh and the parameter-fitting algorithm. The calculated fidelities for the remaining representative points presented in our study are as follows:

- ($p = 1.0\pi, q = 0.5\pi$): 99.34%
- ($p = 0.1\pi, q = 0.99\pi$): 98.89%
- ($p = 1.85\pi, q = 0.3\pi$): 99.64%
- ($p = 0.5\pi, q = 0.5\pi$): 98.83%
- ($p = 1.4\pi, q = 0.9\pi$): 99.88%
- ($p = 0.75\pi, q = 0.8\pi$): 99.13%

These comprehensive results demonstrate that our experimental platform and reconstruction techniques can reliably retrieve the underlying non-Hermitian Hamiltonians across both gapless (Fermi arc) and gapped regions of the non-orientable topology.

S7 Advances and Limitations of Parameter-Space Emulation vs. Lattice-Based Hamiltonians

To comprehensively contextualize our work within the recent advancements in non-Hermitian photonics, we provide a theoretical comparison between our pure parameter-space (p, q) emulation and real-space lattice-based emulations [7, 9, 13, 11, 12].

1. Real-Space Lattice Hamiltonians vs. Parameter-Space Hamiltonians In a typical 1D or 2D real-space emulation, one type of a non-Hermitian system is constructed using an array of coupled physical resonators or waveguides. The Hamiltonian is typically described by a tight-binding model:

$$H_{\text{lattice}} = \sum_{\langle i,j \rangle} (t_{ij}\hat{c}_i^\dagger \hat{c}_j + t_{ji}\hat{c}_j^\dagger \hat{c}_i) + \sum_i (i\gamma_i)\hat{c}_i^\dagger \hat{c}_i \tag{S49}$$

where $t_{ij} \neq (t_{ji})^*$ induces non-reciprocal hopping, and the real parameter γ_i represents site-dependent gain/loss. By invoking periodic boundary conditions (PBC), applying the Fourier transform yields the bulk momentum-space Hamiltonian $\mathcal{H}(\mathbf{k})$.

In our work, instead of building a physical multi-site lattice, we directly emulate the bulk Hamiltonian in the parameter space, $\mathcal{H}(p, q)$, defined on a single structural unit. The time evolution $T(t) = e^{-i\mathcal{H}(p,q)t}$ is synthesized using a fully programmable multi-port unitary dilation mesh.

2. Advance: Unconstrained Topological Flexibility The primary advance of our parameter-space emulation lies in its ability to effortlessly construct non-orientable topological manifolds. To realize a Klein bottle manifold in a physical lattice (Eq. S49), one must enforce a non-symmorphic, twisted boundary condition corresponding to $\mathcal{H}(p,q) = \mathcal{H}(-p,q+\pi)$. In real space, this translates to highly non-local and complex hopping amplitudes connecting distant sites across the lattice, which is physically prohibitive in most integrated photonic platforms. Conversely, by varying the controlling parameters (e.g., MZI phases θ, ϕ) in our programmable PIC to map the mathematical domain $(p,q) \in [-\pi,\pi]\times[0,\pi]$ with the requisite boundary identifications, we can synthesize these topological environments exactly, enabling the direct experimental mapping of the corresponding exceptional points and Fermi arcs.

3. Limitation: Absence of Open Boundary Dynamics A fundamental limitation of our (p,q) space emulation is the absence of real-space physical boundaries. In real-space lattice emulations under open boundary conditions (OBC), the interplay between non-reciprocal hopping and boundaries often leads to the non-Hermitian skin effect (NHSE). In such systems, an extensive number of eigenstates collapse onto the boundaries, with the spatial wavefunction decaying exponentially:

$$|\psi(x)| \propto \left|\sqrt{\frac{t_{\mathrm{L}}}{t_{\mathrm{R}}}}\right|^{x} \tag{S50}$$

where t_{R} and t_{L} are asymmetric right- and left-hopping rates. Because our emulation inherently probes the bulk momentum space point-by-point, there is no spatial coordinate x or physical edge. As a result, boundary-induced phenomena, such as topological edge states or the anomalous wavepacket pile-up of the NHSE, cannot be directly visualized or transported across our circuit with this setup. Thus, real-space lattice emulations and our parameter-space emulations serve as highly complementary paradigms: the former excels at boundary dynamics, while the latter unlocks synthetic manifolds and precise bulk operator reconstruction.

S8 Further Discussion

S8.1 Perspectives on Quantum Photonics and Engineered Loss in PIC

As noted in the main text, while our current demonstration focuses on classical laser light, the programmable non-Hermitian platform is inherently compatible with quantum photonic applications. The problems regarding lossy circuits in quantum optics typically pertain to uncontrolled, passive background loss (e.g., scattering or absorption), which introduces vacuum noise and degrades quantum coherence.

In our architecture, however, the non-Hermitian evolution is realized through *engineered loss*, where signal modes are coupled to ancilla modes via programmable MZIs. This approach offers two distinct advantages for quantum experiments:

1. **Ancilla-Assisted Quantum Measurements:** The "lossy" part of the Hamiltonian is implemented by diverting photons into ancilla ports. Instead of being lost to the environment, these photons can be routed to superconducting nanowire single-photon detectors (SNSPDs). This allows for conditional quantum operations and the study of quantum trajectories under continuous measurement.

2. **Coherent Control of Quantum Light:** Recent work using a similar programmable architecture has demonstrated the emulation of *coherent absorption* of quantum light [4]. In such experiments, engineered loss acts as a coherent gate that enables phase-dependent control over single-photon and NOON-state probabilities. For instance, the transition from perfect transmission to perfect

absorption of a quantum state can be achieved by precisely tuning the coupling to the ancilla modes:

$$\hat{a}_{\text{out}} = \cos(\theta)\hat{a}_{\text{in}} + i\sin(\theta)\hat{a}_{\text{ancilla}} \tag{S51}$$

where the $\hat{a}_{\text{ancilla}}$ mode is initially in the vacuum state or a controlled auxiliary state.

Thus, the programmability of our PIC ensures that the "loss" is a functional resource for quantum state manipulation rather than a parasitic limitation, opening avenues for exploring non-Hermitian topological protection in multi-photon systems.

S8.2 Braid Group Structure and Anyons

The topological charges characterizing exceptional points (EPs) in our system are elements of the braid group B_n. In our two-band system, the fusion behavior we observe—where the system's only two EPs merge into a monopole with charge σ_1^2 rather than annihilating—is a direct consequence of the braid group structure on the Klein-bottle topology, where the total winding number can be non-zero. On the torus, or any other orientable manifold, the sum of all EPs' winding numbers is always zero. For systems with two bands this means that if there are only two EPs, they must fuse to $\sigma_1 \cdot \sigma_1^{-1} = 1$ and annihilate. Creating interesting monopoles in orientable systems thus requires three or more bands, where the non-abelian nature of braids can be exploited to fuse to $\sigma_1\sigma_2^{-1}$, for example, which is non-trivial with total winding number 0.

The braid-group structure of exceptional-point charges also suggests a formal analogy to anyonic braiding. In the present setting, however, these charges live in parameter space rather than describing mobile quasiparticles in real space, and for our two-band model the invariant reduces to an integer winding number. We therefore regard the connection to anyons as an interesting mathematical analogy and a possible future direction, rather than a literal realization of synthetic anyons in the present experiment.

The programmable nature of our platform enables such future investigations of EPs as analogue anyons, as well as geometric phases accumulated during parameter-space braiding operations.

References

[1] Christopher Alexiev et al. "Calibrating rectangular interferometer meshes with external photodetectors". In: *OSA Continuum* 4.11 (2021), pp. 2892–2904. DOI: 10.1364/OSAC.437918.

[2] Léon Autonne. *Sur les matrices hypohermitiennes et sur les matrices unitaires*. A. Rey, 1915.

[3] Xianhui Fu et al. "Non-Hermitian systems based on 3D chirality enabled asymmetrical polarization switching and omni-polarizer action at an EP". In: *Light: Science & Applications* 14.1 (2025), p. 383. DOI: 10.1038/s41377-025-01960-5.

[4] Govind Krishna et al. "Emulation of Coherent Absorption of Fock-State Quantum Light in a Programmable Linear Photonic Circuit". In: *Nature Communications* 17 (2026), p. 4211. DOI: 10.1038/s41467-026-72850-6.

[5] Shuqing Lin et al. "Power-efficient programmable integrated multiport photonic interferometer in CMOS-compatible silicon nitride". In: *Photonics Research* 12.3 (2024), A11–A20. DOI: 10.1364/PRJ.507548.

[6] Hadiseh Nasari et al. "Observation of chiral state transfer without encircling an exceptional point". In: *Nature* 605.7909 (2022), pp. 256–261. DOI: 10.1038/s41586-022-04542-2.

[7] Fahri Emre Öztürk et al. "Observation of a non-Hermitian phase transition in an optical quantum gas". In: *Science* 372.6537 (2021), pp. 88–91. DOI: 10.1126/science.abe9869.

[8] Orr Moshe Shalit. “Dilation theory: a guided tour”. In: *Operator theory, functional analysis and applications*. Vol. 282. Operator Theory: Advances and Applications. Springer, 2021, pp. 551–623. DOI: 10.1007/978-3-030-51945-2_28.

[9] Wange Song et al. “Observation of Weyl interface states in non-Hermitian synthetic photonic systems”. In: *Physical Review Letters* 130.4 (2023), p. 043803. DOI: 10.1103/PhysRevLett.130.043803.

[10] Teiji Takagi. “On an algebraic problem reluted to an analytic theorem of carathéodory and fejér and on an allied theorem of landau”. In: *Japanese Journal of Mathematics: Transactions and Abstracts* 1 (1924), pp. 83–93. DOI: 10.4099/jjm1924.1.0_83.

[11] Guo-Huai Wang et al. “Coupled non-Hermitian skin effect with exceptional points”. In: *Light: Science & Applications* 14.1 (2025), p. 339. DOI: 10.1038/s41377-025-02006-6.

[12] Rui Ye et al. “Observing non-Hermiticity induced chirality breaking in a synthetic Hall ladder”. In: *Light: Science & Applications* 14.1 (2025), p. 39. DOI: 10.1038/s41377-024-01700-1.

[13] Xinyuan Zheng et al. “Dynamic control of 2D non-Hermitian photonic corner skin modes in synthetic dimensions”. In: *Nature Communications* 15.1 (2024), p. 10881. DOI: 10.1038/s41467-024-55236-4.
